# The Association between the Levels of Serum Ferritin and Sex Hormones in a Large Scale of Chinese Male Population

**DOI:** 10.1371/journal.pone.0075908

**Published:** 2013-10-11

**Authors:** Zhenfang Liu, Fanghui Ye, Haiying Zhang, Yong Gao, Aihua Tan, Shijun Zhang, Qiang Xiao, Bing Zhang, Lulu Huang, Bingbing Ye, Xue Qin, Chunlei Wu, Zheng Lu, Youjie Zhang, Ming Liao, Xiaobo Yang, Zengnan Mo

**Affiliations:** 1 Hematology Department, The First Affiliated Hospital of Guangxi Medical University, Nanning, Guangxi, China; 2 Center for Genomic and Personalized Medicine, Guangxi Medical University, Nanning, Guangxi, China; 3 Department of Occupational Health and Environmental Health, School of Public Health, Guangxi Medical University, Nanning, Guangxi, China; 4 Department of Clinical Laboratory, The First Affiliated Hospital of Guangxi Medical University, Nanning, Guangxi, China; 5 Institute of Urology and Nephrology, The First Affiliated Hospital of Guangxi Medical University, Nanning, Guangxi, China; Cardiff University, United Kingdom

## Abstract

**Background:**

The ferritin is an important participant of iron-storage but its regulation and related factors were not well defined. The present objective was to explore the potential association between serum ferritin levels and sex hormones.

**Methods:**

1999 Chinese men in the Fangchenggang Area Male Health and Examination Survey (FAMHES) were recruited in this cross-sectional study. Levels of serum ferritin, total testosterone (free testosterone was calculated from the total one), estradiol and sex hormone-binding protein were detected in venous blood samples. The effects of age, BMI, smoking as well as alcohol consumption were analyzed on ferritin levels, respectively, and then the Pearson’s correlation analysis was used to evaluate the association between ferritin levels and sex hormones adjusting for the above factors.

**Results:**

The age, BMI and alcohol consumption significantly affected serum ferritin levels, but there was no significant difference between smokers and nonsmokers. Ferritin levels were significantly and negatively associated with total testosterone (*R* = −0.205, *P*< 0.001), sex hormone-binding protein (*R* = −0.161, *P*<0.001) and free testosterone (*R* = −0.097, *P*<0.001). After age and alcohol consumption were adjusted, the above associations were still significant (*R* = −0.200, −0.181 and −0.083, respectively, all *P*<0.001). However, there was only borderline negative association between ferritin levels and estradiol (adjusted *R* = −0.039, *P* = 0.083).

**Conclusion:**

The large scale of epidemic results showed the significantly negative associations between serum ferritin levels and sex hormones, which may provide more clues to explore the potential regulation and biological mechanism of ferritin.

## Introduction

Ferritin is a protein that contributes to the iron-storage mainly in our body and distributes widely in all tissues, especially in some organs such as the liver, spleen and bone marrow [1], and it is also an inflammatory serum biomarker [2]. Some previous studies have demonstrated that levels of ferritin were related to several disease states. As we knew, it was associated with metabolic syndrome [3],hematopoiesis [4], [5], renal disease [6], [7], liver disease [8], autoimmunity [9], infection [10], the prognosis of cancer [11]and the mortality of liver transplantation [12].

It was reported that the two subunits of ferritin was synthesized under the control of different genes in chromosomes 11 and 19, respectively [13], [14]. The level of ferritin was affected by the iron metabolism in humans body and cytokines, growth factor as well as oxidants contributed to the regulation [15]. Especially, it should be noted that the hormones also may be a participant in the regulation, for example the thyroid hormone [15]. When it came to the sex hormones, they were well known as important participants in improving sexual organs, and also related to metabolic syndrome [16], anemia [17], inflammation [18] and the mortality in the elderly [19]–[21].

In view of common diseases and the regulation mechanism of ferritin that were mentioned above, sex hormones may potentially take part in the regulation of ferritin, and then emerge some evidences of correlations between them and serum ferritin levels. However, few studies did describe the relationships between serum ferritin levels and sex hormones [22], [23], and we were the first to perform a large scale of epidemiological investigation to directly explore the clues of their correlation.

## Subjects and Methods

### Study design and subjects recruitment

The Fangchenggang Area Male Health and Examination Survey (FAMHES) was designed to investigate the effects that environmental and genetic factors and their interaction had on the development of age-related chronic diseases and the people who participated in this investigation was non-institutionalized men from 17 to 88 years old in Guangxi, China. A total number of 4303 continuous participants in a large-scale physical examination in the Medical Center of Fangchenggang First People’s Hospital from September 2009 to December 2009 were under a comprehensive demographic and health survey. All trained interviewers used this questionnaire to collect epidemiological data through face- to-face interview following the structured guidelines in condition of obtaining written informed consent and the Ethics and Human Subjects Committee of the Guangxi Medical University did approve of this study. Of the 4303 participants, 3136 was self-reported pure Han nationality for three generations and Fangchenggang residents. We excluded the participants with the self-reported information of neoplastic condition, diagnosis of chronic disease, infection with acute infectious diseases, use of any drug with a known effect on the endocrine system or who was considered as prescribed in the past two weeks and people whose data was incomplete about smoking and alcohol consumption were also excluded. Thus, 1999 men was finally recruited and then 10.0 ml venous blood samples was collected from everyone [24].

### Survey of BMI and living habits

In the present study, age, body mass index (BMI), smoking, alcohol consumption were considered as potential confounding predictors for ferritin and/or sex hormones [25], [26]. Height and weight were obtained with participants with light clothing and without shoes, and in this study the value of 25 was treated as the threshold for BMI. Conditions of smoking and alcohol consumption were self-reported, participants who had never smoked or had stopped smoked for 6 months as least to the time for interview were considered as ‘nonsmoker’, while the people who had smoked for 6 months or longer and still smoked to the time for interview or had stopped smoked less than 6 months were ‘smokers’. For alcohol consumption, participants who had ever consumed three or more drinks (beer, wine, and hard liquor) weekly and done so for six consecutive months were marked with ‘yes’ as described elsewhere [27].

### Detection of serum ferritin and sex hormones

Ferritin, total testosterone, sex hormone-binding protein (SHBG) and estradiol were measured with electrochemiluminescence immunoassay on COBAS 6000 system E601 (Elecsys module) immunoassay analyzers (Roche Diagnostics, IN, Germany) with the same batch of reagents. The interassay coefficients of variation were 3.4%, 3.6%, 4.4% and 3.4%, respectively. The free testosterone (FT) and free testosterone index (FTI) were calculated from the detected total testosterone by the described procedure [28].

### Statistical analyses

Basic characteristics and information of variables were shown by Mean±Standard Deviation (SD), data of serum total testosterone, free testosterone, estradiol and SHBG were normally distributed. Additionally, data of serum ferritin was shown by median and interquartile range, and it was normally distributed after logarithmic transformation (log-ferritin). The One-way ANOVA was used to make comparison in subgroups of age, BMI, smoking and alcohol consumption for log-ferritin. Especially, the multiple comparisons between subgroups were carried under Fisher's LSD method. The analysis of Pearson’s correlation coefficient and Partial correlation coefficient were performed to explore the correlations between log-ferritin and other variables respectively, and the latter was in condition of age and alcohol consumption were adjusted because BMI was correlated with ferritin and age. Besides, the quartile of serum total testosterone, free testosterone and SHBG was obtained and the correlation between them and relevant log-ferritin was explored respectively. All the analysis was performed by SPSS 17.0.

## Results

### General information of the 1999 Chinese men

Basic characteristics and information of variables were shown in Table 1. A total number of 1999 men were recruited in the present study with the age of 37.54±11.10 years (aged 20–69 years) and the BMI of 23.30±3.35 kg/m^2^. Data of serum ferritin was not normally distributed with the result of 318.00 (221.40, 459.90) ng/ml and then it was under the logarithmic transformation for further analysis (2.49±0.27 ng/ml, 95%CI: 1.25–2.89). The levels of serum free testosterone (0.42±0.12 nM, 95%CI: 0.14–0.62) and free testosterone index (58.28±22.58%) were calculated from the data of detected total testosterone (6.27±1.91 ng/ml, 95%CI: 2.20–9.70). In addition, 590 subjects (29.5%) were considered as “overweight”, and 1021 subjects (51.1%) were considered as “smoker” and 1651 participants (82.6%) were marked with “yes” in view of alcohol consumption.

**Table 1:** Characteristics and variables of the 1999 men who conformed well this study.

Variable	Mean±SD
Age (year)	37.54±11.10
BMI (kg/m^2^)	23.30±3.35
Ferritin^a^ (ng/ml)	318.00 (221.40, 459.90)
Estradiol (pg/ml)	34.44±9.99
SHBG^b^ (nM)	42.21±20.56
Total testosterone (ng/ml)	6.27±1.91
Free testosterone (nM)	0.42±0.12
Free testosterone index (%)	58.28±22.58

Ferritin^a^: the data was shown by median and interquartile range;

SHBG^b^: sex hormone-binding globulin.

### Serum ferritin in subgroups based on general information

Comparisons for levels of serum ferritin in different subgroups were performed based on age, BMI, smoking and alcohol consumption (Table 2). Our results showed that age was significantly associated with levels of serum ferritin with *P*<0.001, especially, and those male subjects in the age subgroup of 40–49 years had the highest serum ferritin levels (2.53±0.25 ng/ml), but no significant trend was found with the increasing age groups, *P*>0.05. When the value of 25 kg/m^2^ was considered as the threshold on the BMI stratification, a significant difference was also shown between two subgroups (2.45±0.28 vs 2.59±0.23 ng/ml) with *P*<0.001. In addition, the alcohol consumption was also significantly associated with levels of serum ferritin and those subjects who were marked with “yes” had higher ferritin levels than those marked with “no” (2.50±0.27 vs 2.46±0.28 ng/ml, *P* = 0.011), however, no significant difference was found between smokers and nonsmokers (2.50±0.27 vs 2.49±0.27 ng/ml, *P* = 0.447).

**Table 2:** Comparisons about the concentration of serum ferritin based on general information that stratified by subgroups.

Variable	Number (%)	Serum ferritin^a^ (ng/ml)	*P*-value
Age (year)			<0.001
20–29	556 (27.81)	2.44±0.24	
30–39	682 (34.12)	2.52±0.26	
40–49	474 (23.71)	2.53±0.25	
50–59	172 (8.60)	2.47±0.34	
60–69	115 (5.75)	2.47±0.36	
BMI (kg/m^2^)			<0.001
<25	1409 (70.49)	2.45±0.28	
≥25	590 (29.51)	2.59±0.23	
Smoking			0.447
yes	1021 (51.08)	2.50±0.27	
no	978 (48.92)	2.49±0.27	
Alcohol consumption			0.011
yes	1651 (82.59)	2.50±0.27	
no	348 (17.41)	2.46±0.28	

Serum ferritin^a^: the data of serum ferritin was log-transformed and the results were present by Mean ± SD.

### Correlation between serum ferritin and sex hormone/sex hormone-binding protein

We firstly investigated the direct correlation between ferritin and sex hormone levels without any adjustment of potential confounding factors. Our data showed that the correlation between serum log-ferritin and all sex hormones were significant and negative (Table 3), including total testosterone, free testosterone and SHBG, all *P*<0.001(*R*  = –0.205, –0.097 and –0.161, respectively), and the correlation between serum ferritin and estradiol levels was also significant (*R* = –0.048, *P* = 0.034). Furthermore, the analysis of covariate correlation was used to explore the association between them after the adjustment of potential confounding factors (age and alcohol consumption), and similarly significant results were obtained (Table 3), the correlations between ferritin and total testosterone, free testosterone and SHBG were still significant (*R* = –0.200, –0.083 and –0.182, respectively), all *P*<0.001. However, the correlation was not significant any longer between ferritin and estradiol level (*R* = –0.039, *P* = 0.083).

**Table 3:** The correlations between serum log-ferritin (ng/ml) and other detected variables.

Variables	*R_1_* ^a^	*P*-value	*R_2_* ^b^	*P*-value
Total testosterone (ng/ml)	–0.205	<0.001	–0.200	<0.001
Free testosterone (nM/L)	–0.097	<0.001	–0.083	<0.001
SHBG^c^ (nM/L)	–0.161	<0.001	–0.182	<0.001
Estradiol (pg/ml)	–0.048	0.034	–0.039	0.083

R_1_^a^_:_ Pearson’s correlation coefficient in condition of none was adjusted; R_2_^b^_:_ Partial correlation coefficient in condition of age and alcohol consumption was adjusted; SHBG^c^: sex hormone-binding globulin;

Additionally, we stratified all subjects into four subgroups according to the quartiles of sex hormone levels, and those subjects with the free testosterone levels from 0.42 to 0.47 nM had higher ferritin levels than those with levels from 0.35–0.41 nM, *P*<0.001. Especially, we also found a significant decrease trend of ferritin levels with the rise of total testosterone (*R* = –0.997, *P*<0.001), free testosterone (*R* = –0.838, *P*<0.001) and SHBG (*R* = –0.983, *P*<0.001).

## Discussion

In our population-based cross-sectional study, data showed that levels of serum ferritin were negatively associated with sex hormones. For serum total testosterone, free testosterone and SHBG, the associations were significant whether those potential confounding factors were adjusted or not.

The small quantity of human serum ferritin was regulated by several factors and associated with several diseases [3], [4], [15], [29]. Previous reports revealed that Tumor necrosis factor [30], inflammation [31], thyroid hormone [32], B-boxing-factor [33] and insulin [34] may regulate the ferritin levels in different sides. Besides, abnormal functions of serum ferritin would lead to the iron deficiency anemia [4], [5], age-related cataract [29] and cardiovascular diseases [3]. Above all, the regulation of serum ferritin is divers. Meanwhile, it may be an important multiple-perspective regulator in humans through many biochemical and molecular mechanisms that remain to explore. For testosterone, as we all knew it was one of the most active androgens and mainly bounded to SHBG and albumin. However, only free testosterone was bioactive [35], [36]. So far we know that testosterone was with important functions of regulatory [37] and also related to many diseases, such as anemia, metabolic syndrome and the iron stage [18], [36]. Furthermore, previous studies had also showed that the levels of total testosterone and SHBG were negatively associated with metabolic syndrome [16] while the levels of serum ferritin was in a positive association with metabolic syndrome [3]. In prostate cancer, lower testosterone or higher ferritin levels was related with poorer diagnosis or estimation relatively [38]–[40]. Besides, serum ferritin was considered as a risk factor [3] while the sex hormone may benefit for women in cardiovascular diseases [22]. And the mentioned above was consistent with our results.

Interestingly, the correlation between serum ferritin and sex hormones was value of a deeper exploration in term of hematopoiesis. For erythrocytosis, it was significantly related with sex hormones, especially the testosterone, because the iron was stimulated into erythrocyte by testosterone [41]–[44] and the process was under a strong regulation of hepcidin [45]–[47]. Reports also showed that humans were with a gender difference in lower limits of haemoglobin, serum ferritin, and red blood cell count [18] and the difference in haemoglobin at puberty might be owing to testosterone increased the upper limit of haemoglobin in humans [41]. However, testosterone was suppressed by hepcidin and it would lead to more serum free iron while estradiol could suppress the hepcidin which usually led to the increasing iron uptake [7], [48].On the other hand, ferritin and transferrin were in a significant relation with iron metabolism and they were under the control of hepcidin [48] due to hepcidin was positively associated with levels of serum ferritin and transferrin receptor [45]. Nevertheless, in order to gain an object and convictive cognize of the relation between ferritin and sex hormones we should be paid more attention to the micro parts.

To our knowledge, there was no direct investigation to explore relationships between sex hormones [18] and iron storage before and our data showed that those associations were negative. In addition, it was well known that the ferritin L and H genes were located in 11q12 and 19q13 respectively [13], [14]. Moreover, the genes *GSTP1* and *SLCO2B1*in 11q13 as well as the genes *LHB* and *KLK3*in 19q13were reported to involve in sex hormones metabolic pathway, though the previous study showed that the latter two genes were not so significant for risk factor in prostate cancer [49]. It should be noted that the gene *GSTP1* was in an association with ferritin H gene [50], gene *LHB* was significantly with testosterone and estradiol levels [51] and special *CYP3A/KLK3* genotypes increased metastatic disease while the expression of *CYP3A* in prostate cancer was regulated by androgen [52], [53]. Considering the diseases with abnormal levels of serum testosterone and ferritin, adding those genes mentioned above were in such a near location, it seems that the potential interact effect between serum ferritin levels and sex hormones is probable. Exceptionally, some reports revealed that the liver iron overload related with higher concentration of SHBG [54] and the Fe-deficient diet did not affect the testosterone in adult male rat in a relatively short time [23].

Some study limitations needed to be addressed when interpreting these results. Firstly, this present study was a cross-sectional design so that the direction and convincingness of association should be validated by some experiments *in vitro*. Secondly, the female subjects were not included in the participants so that we could not make a comparison in gender and the results may lack of enough persuasion. Thirdly, there was no enough data about iron metabolism obtained in the present study so that we could not make more comprehensive analyses of the association between the serum ferritin and sex hormones.

## Conclusion

In conclusion, our data showed significantly negative associations between levels of serum ferritin and sex hormones, and these results may be helpful to the further exploration about the regulation or mechanisms of the ferritin.

## References

[1] Worwood M, Aherne W, Dawkins S, Jacobs A (1975) The characteristics of ferritin from human tissues, serum and blood cells. Clin Sci Mol Med 48: 441–451. 1168559 10.1042/cs0480441

[2] Jones BM, Worwood M, Jacobs A (1980) Serum ferritin in patients with cancer: determination with antibodies to HeLa cell and spleen ferritin. Clin Chim Acta 106: 203–214. 6250746 10.1016/0009-8981(80)90173-4

[3] Yoo KD, Ko SH, Park JE, Ahn YB, Yim HW, et al (2012) High serum ferritin levels are associated with metabolic risk factors in non-obese Korean young adults: Korean National Health and Nutrition Examination Survey (KNHANES) IV. Clin Endocrinol (Oxf) 77: 233–240. 21977991 10.1111/j.1365-2265.2011.04248.x

[4] Przybyszewska J, Zekanowska E, Kedziora-Kornatowska K, Boinska J, Cichon R, et al (2013) Serum prohepcidin and other iron metabolism parameters in elderly patients with anemia of chronic disease and with iron deficiency anemia. Pol Arch Med Wewn 123: 105–111. 23299925 10.20452/pamw.1623

[5] Choi HS, Song SH, Lee JH, Kim HJ, Yang HR (2012) Serum hepcidin levels and iron parameters in children with iron deficiency. Korean J Hematol 47: 286–292. 23320008 10.5045/kjh.2012.47.4.286 PMC3538801

[6] Brockenbrough AT, Dittrich MO, Page ST, Smith T, Stivelman JC, et al (2006) Transdermal androgen therapy to augment EPO in the treatment of anemia of chronic renal disease. Am J Kidney Dis 47: 251–262. 16431254 10.1053/j.ajkd.2005.10.022

[7] Bachman E, Feng R, Travison T, Li M, Olbina G, et al (2010) Testosterone suppresses hepcidin in men: a potential mechanism for testosterone-induced erythrocytosis. J Clin Endocrinol Metab 95: 4743–4747. 20660052 10.1210/jc.2010-0864 PMC3050108

[8] Tan TC, Crawford DH, Franklin ME, Jaskowski LA, Macdonald GA, et al (2012) The serum hepcidin:ferritin ratio is a potential biomarker for cirrhosis. Liver Int 32: 1391–1399. 22676252 10.1111/j.1478-3231.2012.02828.x

[9] Zandman-Goddard G, Shoenfeld Y (2008) Hyperferritinemia in autoimmunity. Isr Med Assoc J 10: 83–84. 18300583

[10] Ratledge C (2007) Iron metabolism and infection. Food Nutr Bull 28: S515–523. 18297890 10.1177/15648265070284S405

[11] Alkhateeb AA, Leitzel K, Ali SM, Campbell-Baird C, Evans M, et al (2012) Elevation in inflammatory serum biomarkers predicts response to trastuzumab-containing therapy. PLoS One 7: e51379. 23300545 10.1371/journal.pone.0051379 PMC3530544

[12] Walker NM, Stuart KA, Ryan RJ, Desai S, Saab S, et al (2010) Serum ferritin concentration predicts mortality in patients awaiting liver transplantation. Hepatology 51: 1683–1691. 20225256 10.1002/hep.23537

[13] Caskey JH, Jones C, Miller YE, Seligman PA (1983) Human ferritin gene is assigned to chromosome 19. Proc Natl Acad Sci U S A 80: 482–486. 6572903 10.1073/pnas.80.2.482 PMC393402

[14] Worwood M, Brook JD, Cragg SJ, Hellkuhl B, Jones BM, et al (1985) Assignment of human ferritin genes to chromosomes 11 and 19q13.3—19qter. Hum Genet 69: 371–374. 3857215 10.1007/BF00291657

[15] Torti FM (2002) Regulation of ferritin genes and protein. Blood 99: 3505–3516. 11986201 10.1182/blood.v99.10.3505

[16] Hong D, Kim YS, Son ES, Kim KN, Kim BT, et al (2013) Total testosterone and sex hormone-binding globulin are associated with metabolic syndrome independent of age and body mass index in Korean men. Maturitas 74: 148–153. 23218685 10.1016/j.maturitas.2012.10.016

[17] Waalen J, von Lohneysen K, Lee P, Xu X, Friedman JS (2011) Erythropoietin, GDF15, IL6, hepcidin and testosterone levels in a large cohort of elderly individuals with anaemia of known and unknown cause. Eur J Haematol 87: 107–116. 21535154 10.1111/j.1600-0609.2011.01631.x

[18] Laaksonen DE, Niskanen L, Punnonen K, Nyyssonen K, Tuomainen TP, et al (2003) Sex hormones, inflammation and the metabolic syndrome: a population-based study. Eur J Endocrinol 149: 601–608. 14641004 10.1530/eje.0.1490601

[19] Tivesten A, Vandenput L, Labrie F, Karlsson MK, Ljunggren O, et al (2009) Low serum testosterone and estradiol predict mortality in elderly men. J Clin Endocrinol Metab 94: 2482–2488. 19401373 10.1210/jc.2008-2650

[20] Wu IC, Lin XZ, Liu PF, Tsai WL, Shiesh SC (2010) Low serum testosterone and frailty in older men and women. Maturitas 67: 348–352. 20727696 10.1016/j.maturitas.2010.07.010

[21] Shores MM, Smith NL, Forsberg CW, Anawalt BD, Matsumoto AM (2012) Testosterone treatment and mortality in men with low testosterone levels. J Clin Endocrinol Metab 97: 2050–2058. 22496507 10.1210/jc.2011-2591

[22] Berge LN, Bonaa KH, Nordoy A (1994) Serum ferritin, sex hormones, and cardiovascular risk factors in healthy women. Arteriosclerosis, Thrombosis, and Vascular Biology 14: 857–861. 10.1161/01.atv.14.6.857 8199174

[23] Intragumtornchai T, Steiner RA, Finch CA (1988) Iron deficiency: effect on plasma luteinizing hormone and testosterone levels in the adult male rat. Am J Clin Nutr 48: 641–644. 3414578 10.1093/ajcn/48.3.641

[24] Li S, Lin L, Mo Z, Qin X, Lv H, et al (2011) Reference values for serum ferritin in Chinese Han ethnic males: results from a Chinese male population survey. Clin Biochem 44: 1325–1328. 21907192 10.1016/j.clinbiochem.2011.08.1137

[25] Mohr BA, Bhasin S, Link CL, O'Donnell AB, McKinlay JB (2006) The effect of changes in adiposity on testosterone levels in older men: longitudinal results from the Massachusetts Male Aging Study. Eur J Endocrinol 155: 443–452. 16914599 10.1530/eje.1.02241

[26] Kelly DM, Jones TH (2013) Testosterone: a vascular hormone in health and disease. J Endocrinol 217: R47–71. 23549841 10.1530/JOE-12-0582

[27] Liao M, Huang X, Gao Y, Tan A, Lu Z, et al (2012) Testosterone is associated with erectile dysfunction: a cross-sectional study in Chinese men. PLoS One 7: e39234. 22737230 10.1371/journal.pone.0039234 PMC3380865

[28] Vermeulen A, Verdonck L, Kaufman JM (1999) A critical evaluation of simple methods for the estimation of free testosterone in serum. J Clin Endocrinol Metab 84: 3666–3672. 10523012 10.1210/jcem.84.10.6079

[29] Assia N, Goldenberg-Cohen N, Rechavi G, Amariglio N, Cohen Y (2010) Mutation analysis of the ferritin L-chain gene in age-related cataract. Mol Vis 16: 2487–2493. 21139976 PMC2994742

[30] Beutler B, Cerami A (1989) The biology of cachectin/TNF—a primary mediator of the host response. Annu Rev Immunol 7: 625–655. 2540776 10.1146/annurev.iy.07.040189.003205

[31] Santambrogio P, Cozzi A, Levi S, Arosio P (1987) Human serum ferritin G-peptide is recognized by anti-L ferritin subunit antibodies and concanavalin-A. Br J Haematol 65: 235–237. 3828232 10.1111/j.1365-2141.1987.tb02271.x

[32] Leedman PJ, Stein AR, Chin WW, Rogers JT (1996) Thyroid hormone modulates the interaction between iron regulatory proteins and the ferritin mRNA iron-responsive element. J Biol Chem 271: 12017–12023. 8662626 10.1074/jbc.271.20.12017

[33] Bevilacqua MA, Faniello MC, Russo T, Cimino F, Costanzo F (1994) Transcriptional regulation of the human H ferritin-encoding gene (FERH) in G418-treated cells: role of the B-box-binding factor. Gene 141: 287–291. 8163204 10.1016/0378-1119(94)90587-8

[34] Yokomori N, Iwasa Y, Aida K, Inoue M, Tawata M, et al (1991) Transcriptional regulation of ferritin messenger ribonucleic acid levels by insulin in cultured rat glioma cells. Endocrinology 128: 1474–1480. 1999166 10.1210/endo-128-3-1474

[35] Enea C, Boisseau N, Diaz V, Dugue B (2008) Biological factors and the determination of androgens in female subjects. Steroids 73: 1203–1216. 18640139 10.1016/j.steroids.2008.06.009

[36] Chen G, Li S, Dong X, Bai Y, Chen A, et al (2012) Investigation of testosterone, androstenone, and estradiol metabolism in HepG2 cells and primary culture pig hepatocytes and their effects on 17betaHSD7 gene expression. PLoS One 7: e52255. 23300627 10.1371/journal.pone.0052255 PMC3530596

[37] Hammes A, Andreassen TK, Spoelgen R, Raila J, Hubner N, et al (2005) Role of endocytosis in cellular uptake of sex steroids. Cell 122: 751–762. 16143106 10.1016/j.cell.2005.06.032

[38] Garcia-Cruz E, Piqueras M, Ribal MJ, Huguet J, Serapiao R, et al (2012) Low testosterone level predicts prostate cancer in re-biopsy in patients with high grade prostatic intraepithelial neoplasia. BJU Int 110: E199–202. 22257176 10.1111/j.1464-410X.2011.10876.x

[39] Garcia-Cruz E, Piqueras M, Huguet J, Peri L, Izquierdo L, et al (2012) Low testosterone levels are related to poor prognosis factors in men with prostate cancer prior to treatment. BJU Int 110: E541–546. 22584031 10.1111/j.1464-410X.2012.11232.x

[40] Kuvibidila SR, Gauthier T, Rayford W (2004) Serum ferritin levels and transferrin saturation in men with prostate cancer. J Natl Med Assoc 96: 641–649. 15160979 PMC2640669

[41] Rushton DH, Barth JH (2010) What is the evidence for gender differences in ferritin and haemoglobin? Crit Rev Oncol Hematol 73: 1–9. 19394859 10.1016/j.critrevonc.2009.03.010

[42] Shahani S, Braga-Basaria M, Maggio M, Basaria S (2009) Androgens and erythropoiesis: past and present. J Endocrinol Invest 32: 704–716. 19494706 10.1007/BF03345745

[43] Coviello AD, Kaplan B, Lakshman KM, Chen T, Singh AB, et al (2008) Effects of graded doses of testosterone on erythropoiesis in healthy young and older men. J Clin Endocrinol Metab 93: 914–919. 18160461 10.1210/jc.2007-1692 PMC2266950

[44] Gonzales GF, Gasco M, Tapia V, Gonzales-Castaneda C (2009) High serum testosterone levels are associated with excessive erythrocytosis of chronic mountain sickness in men. Am J Physiol Endocrinol Metab 296: E1319–1325. 19318512 10.1152/ajpendo.90940.2008 PMC2692401

[45] Eguchi A, Mochizuki T, Tsukada M, Kataoka K, Hamaguchi Y, et al (2012) Serum hepcidin levels and reticulocyte hemoglobin concentrations as indicators of the iron status of peritoneal dialysis patients. Int J Nephrol 2012: 239476. 23193472 10.1155/2012/239476 PMC3501962

[46] Kemna EH, Kartikasari AE, van Tits LJ, Pickkers P, Tjalsma H, et al (2008) Regulation of hepcidin: insights from biochemical analyses on human serum samples. Blood Cells Mol Dis 40: 339–346. 18023212 10.1016/j.bcmd.2007.10.002

[47] Chen J, Enns CA (2012) Hereditary hemochromatosis and transferrin receptor 2. Biochim Biophys Acta 1820: 256–263. 21864651 10.1016/j.bbagen.2011.07.015 PMC3234335

[48] Yang Q, Jian J, Katz S, Abramson SB, Huang X (2012) 17beta-Estradiol inhibits iron hormone hepcidin through an estrogen responsive element half-site. Endocrinology 153: 3170–3178. 22535765 10.1210/en.2011-2045 PMC3380311

[49] Cunningham JM, Hebbring SJ, McDonnell SK, Cicek MS, Christensen GB, et al (2007) Evaluation of genetic variations in the androgen and estrogen metabolic pathways as risk factors for sporadic and familial prostate cancer. Cancer Epidemiol Biomarkers Prev 16: 969–978. 17507624 10.1158/1055-9965.EPI-06-0767

[50] Iwasaki K, Mackenzie EL, Hailemariam K, Sakamoto K, Tsuji Y (2006) Hemin-mediated regulation of an antioxidant-responsive element of the human ferritin H gene and role of Ref-1 during erythroid differentiation of K562 cells. Mol Cell Biol 26: 2845–2856. 16537925 10.1128/MCB.26.7.2845-2856.2006 PMC1430308

[51] Huhtaniemi IT, Pye SR, Holliday KL, Thomson W, O'Neill TW, et al (2010) Effect of polymorphisms in selected genes involved in pituitary-testicular function on reproductive hormones and phenotype in aging men. J Clin Endocrinol Metab 95: 1898–1908. 20173016 10.1210/jc.2009-2071

[52] Vaarala MH, Mattila H, Ohtonen P, Tammela TL, Paavonen TK, et al (2008) The interaction of CYP3A5 polymorphisms along the androgen metabolism pathway in prostate cancer. Int J Cancer 122: 2511–2516. 18306354 10.1002/ijc.23425

[53] Moilanen AM, Hakkola J, Vaarala MH, Kauppila S, Hirvikoski P, et al (2007) Characterization of androgen-regulated expression of CYP3A5 in human prostate. Carcinogenesis 28: 916–921. 17116727 10.1093/carcin/bgl222

[54] Gautier A, Laine F, Massart C, Sandret L, Piguel X, et al (2011) Liver iron overload is associated with elevated SHBG concentration and moderate hypogonadotrophic hypogonadism in dysmetabolic men without genetic haemochromatosis. Eur J Endocrinol 165: 339–343. 21646287 10.1530/EJE-11-0215

